# Machine Learning–Based Predictive Farmland Optimization and Crop Monitoring System

**DOI:** 10.1155/2020/9428281

**Published:** 2020-05-10

**Authors:** Marion Olubunmi Adebiyi, Roseline Oluwaseun Ogundokun, Aneoghena Amarachi Abokhai

**Affiliations:** Department of Computer Science, Landmark University, Omu-Aran, Kwara State, Nigeria

## Abstract

E-agriculture is the integration of technology and digital mechanisms into agricultural processes for more efficient output. This study provided a machine learning–aided mobile system for farmland optimization, using various inputs such as location, crop type, soil type, soil pH, and spacing. Random forest algorithm and BigML were employed to analyze and classify datasets containing crop features that generated subclasses based on random crop feature parameters. The subclasses were further grouped into three main classes to match the crops using data from the companion crops. The study concluded that the approach aided decision making and also assisted in the design of a mobile application using Appery.io. This Appery.io then took in some user input parameters, thereby offering various optimization sets. It was also deduced that the system led to users' optimization of information when implemented on their farmlands.

## 1. Introduction

Agriculture is vital for the development of the world. We, humans, benefit from agriculture one way or the other, which has made agriculture a key area of study. Farmers will always need information to refer to, most especially when growing crops that are not common in their land or culture [1]. The average farmer has access to crude sources of information such as TV, radio, newspapers, fellow farmers, government agricultural agencies, farm supply, and traders. There is, therefore, a need for a system that allows farmers access to relevant information [1].

Machine learning is among the trending technologies; hence, there exist several technologies and systems that run on a machine learning framework [2]. In recent times, several machine learning systems in agriculture have been tested and created. Research of several machine learning algorithms' effectiveness in agriculture [2] and other application domains has also been conducted and this is because machine learning is a very effective tool for efficient use of resources, prediction, and management, which are needed in agriculture. Machine learning is the ability of an electrical processing system to acquire knowledge and apply that knowledge [2].

The scope of this work is concerned with food crop agriculture and using machine learning to help optimize land for maximal crop yield by efficiently utilizing land resources. Crop yield relies strongly on how effectively the basic land requirements can be utilized; land here refers to topography, soil type, soil nutrients, water content, sunlight, and all such factors related to crop growth on farmable areas.

## 2. Literature Review

Table 1 presents reviews of some existing technologies and work.

## 3. Materials and Methods

### 3.1. Materials

#### 3.1.1. Database/Crop Datasets

The data that populates this database includes the plant growth parameters that were used to form the individual decision trees in the random forest. Such data include irrigation, spacing, nutrient requirements, location, temperature, and other related factors that originated from several trusted databases. This plant growth condition database is designed to help decision making with the machine learning algorithm.

### 3.2. Method

In this work, machine learning applied what had been used to set parameters and embedded it into a dataset on a mobile application. The machine learning algorithm was designed to maximize land proportion. The dataset contains parameters of some inputs that are critical for plant growth. The machine learning algorithm defines the relationship between these input parameters and certain internally stored prediction parameters and provides a solution for the output. The values in the database have been converted to a range system of 0 to 1; the need for conversion to the same range is due to data incoherence; data was derived from different sources and was therefore inconsistent, thus requiring a specific conversion.

#### 3.2.1. Output Layer

All inputs and their respective weighted values were converted to a range system of 0 to 1.

#### 3.2.2. Decision Layer(s)

This consists of layers of decision that help to classify input data into appropriate groups which also helped making decisions and setting parameters.

#### 3.2.3. Output

This is composed of results from classification.

#### 3.2.4. Classification

This entailed defining sets of groups to which a new observation would belong. The aim of data classification here was to divide the crops into classes based on their respective data; these classes are based on crops growing together most efficiently on a given piece of land. The actual classification was carried out using random forests to allow all inputs to be considered multiple times for better accuracy since the algorithm comprises multiple decision trees.

### 3.3. System Design

**Figure 1:**
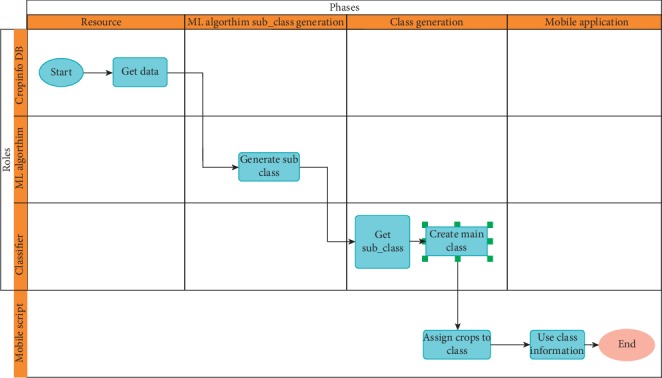
System phases: data flow diagram.

Figure 1 displays the system data flow diagram. The method of classifying and analyzing the results of the classification is divided into phases and functions. The phases include the resource process, which includes the fetching of data from the CropInfo database, which was followed by the generation of machine learning subclasses; the random forest algorithm was used in this phase to create subclasses based on ten different crop feature sets. In the class generation phase, subclasses with similar generation patterns were grouped into three main classes, which are used in the mobile application phase to help optimize the mobile output.

The study also used activity diagrams to analyze the system's behavior and design. This section briefly discusses the interactions between the different activities in the application. It is broken up into three sections:

- The user login activity
- The scheduler
- Tips and tricks activity

#### 3.3.1. The Login Activity Diagram

**Figure 2:**
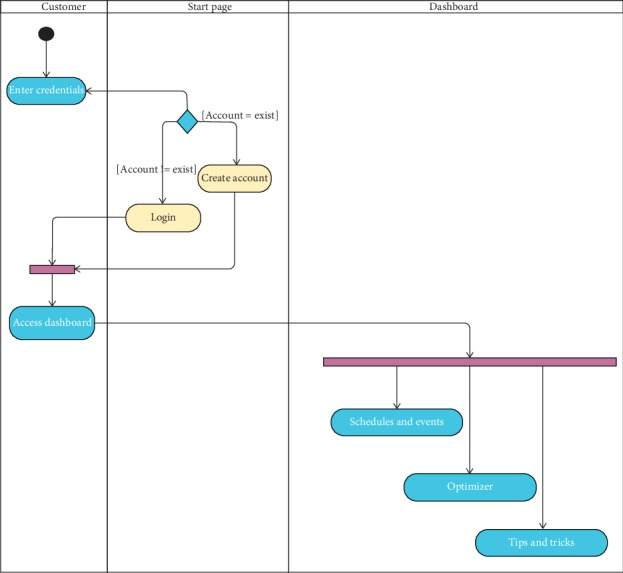
Login activity diagram.

The operation of login as shown in Figure 2 involves a simple user verification process; once user credentials have been submitted, testing will be conducted to decide whether or not the account is valid; when user validity has been verified, the user will have access to the dashboard functions: the key operation, the tips and tricks, and the optimiser.

#### 3.3.2. The Scheduler Activity

**Figure 3:**
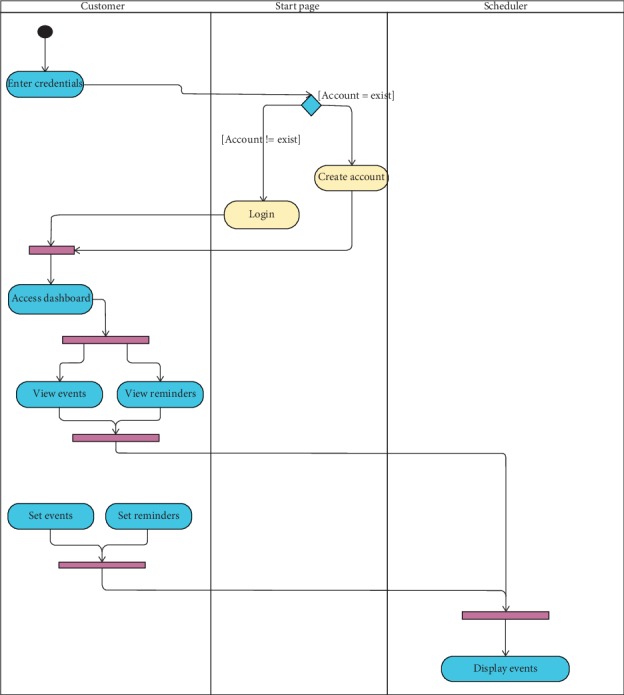
Scheduler activity diagram.

The scheduler activity involves two significant events, as seen in Figure 3. The first one is the schedule event; this task allows users to schedule and display events created by the main task as well as user-generated ones. The second event is the reminder event; this activity allows the user to set reminders and to view active reminders created by the main activity and those created by the user.

#### 3.3.3. The Main Activity

**Figure 4:**
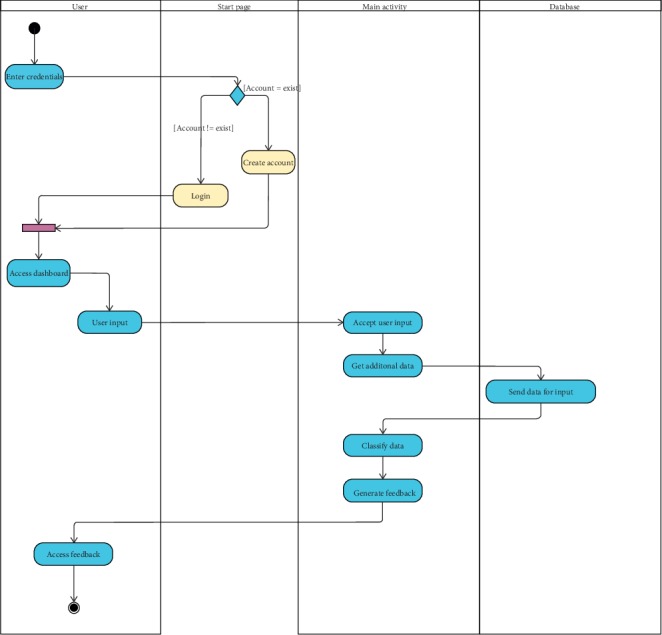
Main activity diagram.

This is the main component of the program, consisting of user input system, machine learning algorithm, feedback system, and database for crop knowledge, as shown in Figure 4.

## 4. Results

The system proposed includes an input collection system incorporating user input, which is processed using the optimiser algorithm. The algorithm used to break down crop features into groups is the random forest. The data from the process is made available as feedback to the customer. This research takes into account the fact that growing crop requirements in Nigeria are not essentially very different from location to location.

### 4.1. Implementation

#### 4.1.1. Classification Model Output

This research uses the random forest classifier to classify the crop resource characteristics into ten subclasses; these subclasses are further categorized into three main classes. The crop, based on the dominant features of a variety, is tailored to its optimum level. In this work, the subclasses of crops were generated using two methods. The first approach involved four random forests generated in BigML; these models were created and analyzed, and results were compared with the performance of the second model, which involved the use of weighted linear equations for decision making. For this classification, these models were used, and each model or tree was used to process the final model. The variance in those models was generated by modifying the model's rules.

OCF represents an ideal match for the class. The weight of each crop feature shall be determined using a set {*x*1, *x*2, ... *x*7 *x*7}. Those sets coincide with a set of values for each weight. Light requirement (Lt), water requirement (W), space requirement (S), location (L), pH requirement (P), soil type (St), and companion (C) are the characteristics to be considered.

#### 4.1.2. Subclass Models: Method One

This study presents four subclass models with two tree samples each, of the ten, that were generated and analyzed as the outputs from each model generation were similar. The parameters for the generation of each of the models were selected randomly, thus varying the output. This was done to allow the fitting of features to certain subclasses if some parameters are absent. These models were used as datasets for designing of the three major classes A, B, and C.

*(1) Model One*. The first model was created using S, Lt, W, and St.

**Figure 5:**
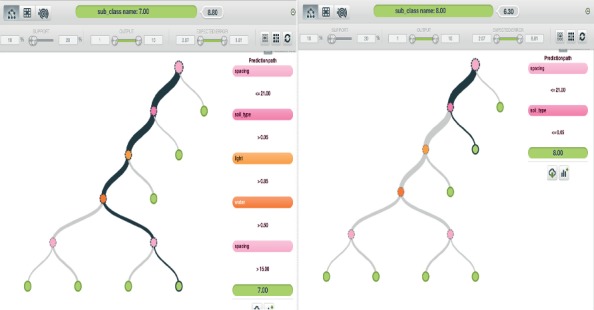
Model one output analysis.

Figure 5 is the subclassification of crops based on the set of random parameters mentioned above, where parameters are not included. This model allowed the classification of crops into their subclasses: location, companion, and pH requirements. This model provided one of the most effective subclass generations of the 10 models that were analyzed.

*(2) Model Two*. The second model was created using S, Lt, W, and L.

**Figure 6:**
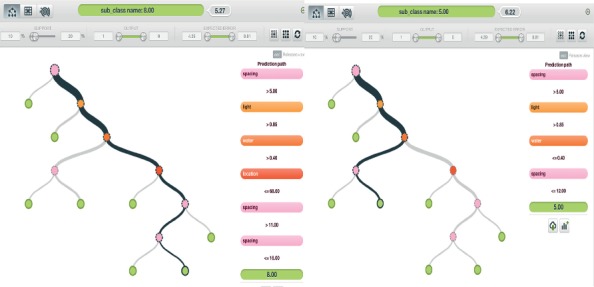
Model two output analysis.

Figure 6 displays the subclass generation solution provided where data are not included for crop pH requirement, soil type, and companions. This was also an efficient model, considering that most plants in the study area essentially need the same type of soil for growth.

*(3) Model Three*. The third model was created using S, L, and Lt.

**Figure 7:**
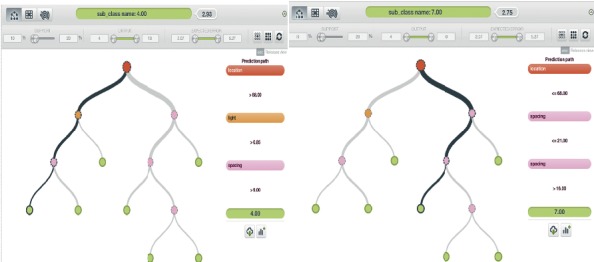
Model three output analysis.

Figure 7 shows model three output analysis, and this model was considered to be the least efficient model of all models considered for subclass generation in terms of the quantity of data used to generate the model, since it provides a subclass generation solution for crops with limited information in the available subclasses.

*(4) Model Four*. The fourth model was created using S, L, and P.

**Figure 8:**
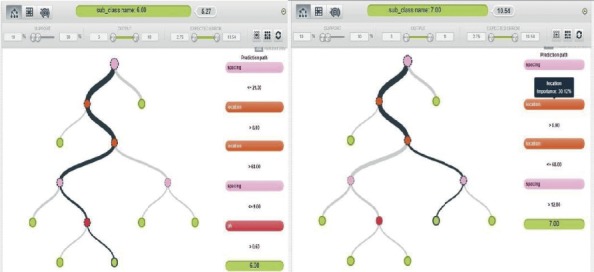
Model four output analysis.

Figure 8 shows that it worked like the third model with a limited number of specified data categories, but it was significantly more efficient than the third model because it includes key parameters that enable the subclass generation to fit more precisely.

#### 4.1.3. Subclass Output: Method Two

After model analysis of method one, it was discovered that, based on the nature of the data, all class generation rules produced similar results due to the uncertainty of the position values; the classification methods below were implemented to create a less ambiguous way to allow for more ideal crop class generations.

*(1) Model One*. Weighted OCF = Lt.*x*5 + W.*x*6 + S.*x*1 + L.*x*2 + P.*x*4 + St.*x*3 + C.*x*7. The weight values for this model's weighted OCF function are fixed values: {1, 1, 2, 4, 6, 8, 10}, where each set value is assigned to the respective weight of the set weights: {*x*1, *x*2, *x*3, *x*4, *x*5, *x*6, *x*7}. This is such as to construct a set of weighted values as follows: waves: {*x*1 = 1, *x*2 = 1, *x*3 = 2, *x*4 = 4, *x*5 = 6, *x*6 = 8, *x*7 = 10}. High functionality is determined for this model from the weighted OCF function, high = {weighted values < 4}, and low function values are determined, low = {weighted values < 4}.

*(2) Model Two*. Weighted OCF = Lt.*x*6 + W.*x*5 + S.*x*2 + L.*x*1 + P.*x*4 + St.*x*3 + C.x7. The weight values for the weighted OCF function for this model are set values: {1, 4, 5, 6, 8, 8, 10}, where each set value is assigned to the respective set weight values: {*x*1, *x*2, *x*3, *x*5, *x*6, *x*7}. This is such as to construct a set of weighted values as follows: waves: {*x*1 = 1, *x*2 = 4, *x*3 = 5, *x*4 = 6, *x*5 = 8, *x*6 = 8, *x*7 = 10}. High functionality is determined for this model from the weighted OCF function, high = {weighted values < 5}, and low function values are determined, low = {weighted values < 5}.

*(3) Model Three*. Weighted OCF = Lt.*x*5 + W.*x*6 + S.*x*1 + L.*x*2 + P.*x*4 + St.*x*3 + C.*x*7. the weight values for the weighted OCF function for this model are represented by the set values: {2, 2, 4, 4, 8, 8, 10}, where each value from the set values is assigned to a respective weight from the set weights: {*x*1, *x*2, *x*3, *x*4, *x*5, *x*6, *x*7}. This is such that set weighted values are created as follows: weighted values: {*x*1 = 2, *x*2 = 2, *x*3 = 4, *x*4 = 4, *x*5 = 8, *x*6 = 8, *x*7 = 10}. For this model, from the weighted OCF function, the high features are determined by the function high = {weighted values ≥ 5}, and the low values are determined by the function low = {weighted values < 5}.

#### 4.1.4. Ideal Class Model Distribution Output

The crop classification efficiency is based on the combined three models. The performance was obtained from the subclass models where similar calculations were made on the output data of the subclass and an additional feature for each subclass to allow fitting to the three final ideal classes; to allow this fitting, accompanying crops were added to the model data. This results in crops with features belonging to class A and class B not interacting, meaning that crops should not be planted together in those groups. The features of class C elements interact with the features of both class A and class B; this means that crops with features of class C can be grown effectively in either of the other two classes.

**Figure 9:**
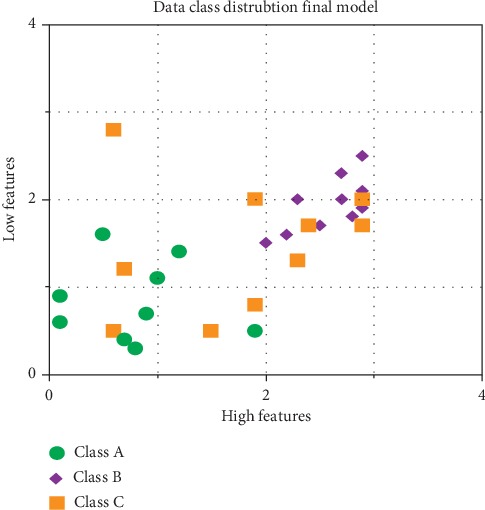
Ideal class distribution.

Based on the distribution and classification of the features shown in Figure 9, the ten crops considered in this work are assigned to their respective classes according to their characteristics as shown in Table 2.

## 5. Discussion

**Figure 10:**
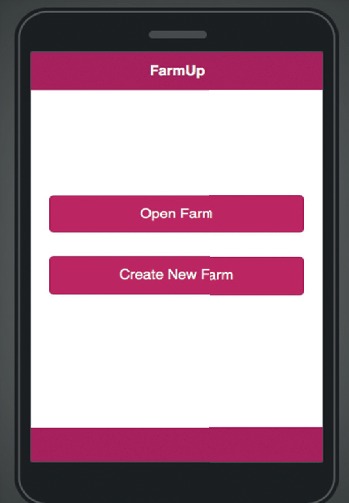
Mobile application start page.

**Figure 11:**
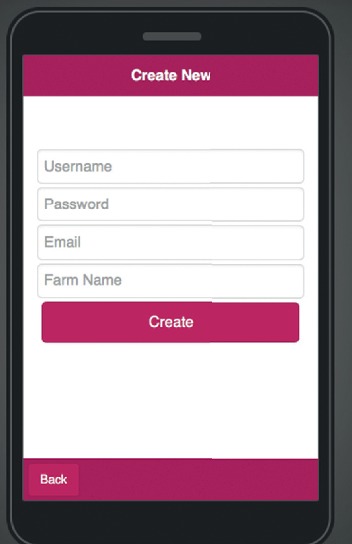
Mobile application “create new farm” page.

**Figure 12:**
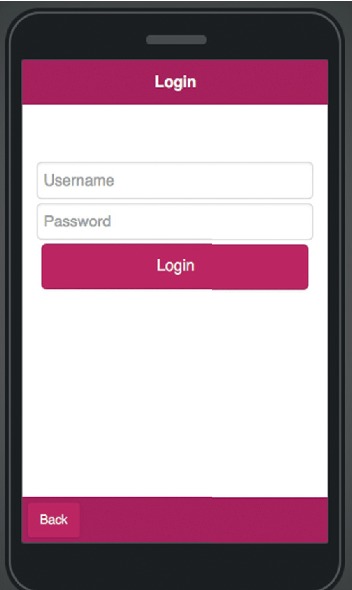
Mobile application “open existing farm” page.

The mobile application allows multiple farm accounts to be opened on the same computer, there are two choices on the start page as shown in Figure 10 to either to create a new farm account as shown in Figure 11 or open an existing farm account as seen in Figure 12.

**Figure 13:**
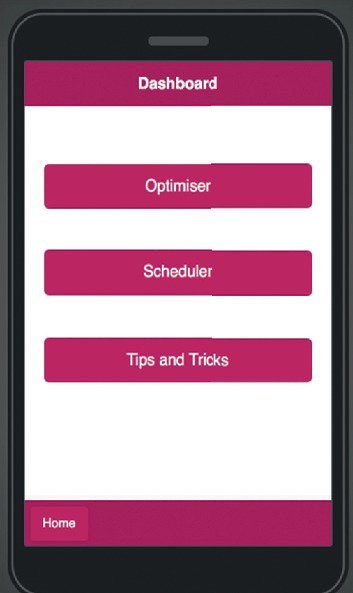
Mobile application dashboard.

**Figure 14:**
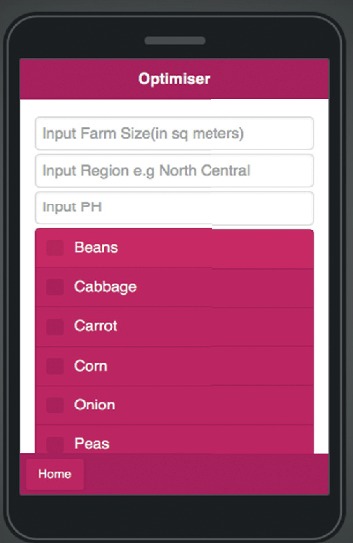
Mobile application optimiser.

The user has access to the dashboard after successful login or sign-up, as shown in Figure 13, and its functions. The functions of the dashboard are the optimiser function, as seen in Figure 14, which is the main application operation; the scheduler function; and the tips and tricks function which contains the knowledge repository.

**Figure 15:**
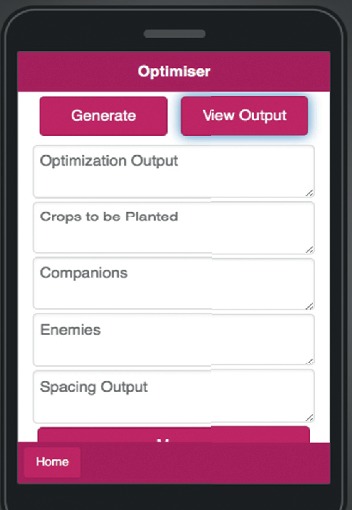
Mobile application optimiser output area.

**Figure 16:**
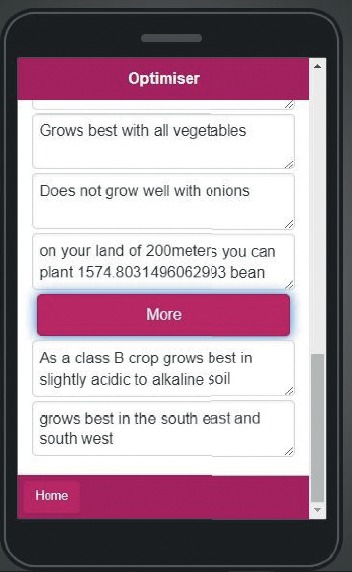
Optimiser with output.

The optimiser consists of three fields of data: the field of farm size input that takes numerical input in square meters, the area field that takes user location input, and the field of pH input that takes the farm soil input as seen in Figure 15. Users choose the crops they want to grow on their farm, and the outputs are displayed in the optimiser output area based on the input as in Figure 16.

**Figure 17:**
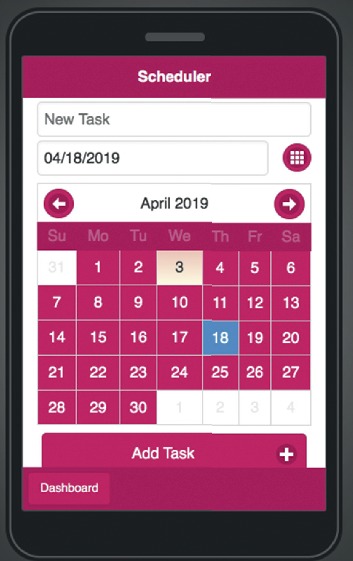
Mobile application scheduler.

The scheduler as seen in Figure 17 enables users to set the events or activities that they wish to perform. The user shall provide the task mark and pick the date of the work to be performed.

**Figure 18:**
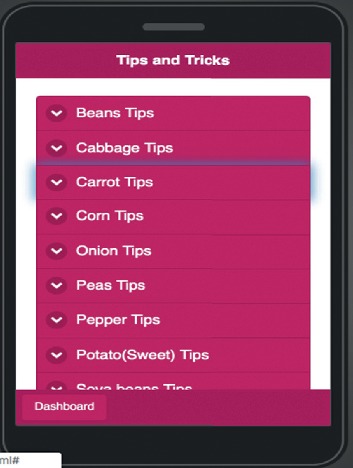
Mobile application tips page.

The tips and tricks event allows users to get agricultural tools as shown in Figure 18. Such tips and tricks are divided into collapsible components; such components include tip tools in the database for each crop and some additional tips on the field. They also include guidelines for pH checking and soil improvement.

## 6. Conclusion

Most farmers do not have access to a central repository of relevant information that will help them make full use of and optimize their farmland. This work provided a mobile application interface that allows farmers to access their farmland information and guarantees them the services they need instantly.

## 7. Future Work

In future work, the machine learning models used to inform parameter setting in the mobile application could be developed using the machine learning algorithm embedded in the system and used to predict.

## Figures and Tables

**Table 1:** Review of existing technologies.

S/N	Author(S)	Year	Problem	Method	Contribution
1	Priya et al. [3]	2018	This work was concerned with the use of the random forest algorithm to generate predictions for crop yield and improvement.	The random forest algorithm was used for yield production using a dataset with four features or parameters. A training set as used to train the algorithm rules which were then applied to the remaining datasets.	The results showed that we can attain an accurate crop yield prediction using the random forest algorithm. Random forest algorithm achieves a largest number of crop yield models with lowest models. It is suitable for massive crop yield prediction in agricultural planning.

2	Jeong et. al. [4]	2016	This work aimed at examining the performance efficiency of the random forest algorithm in crop yield prediction for the wheat crop, potato crop, and maize crop.	The random forest algorithm was used to train the datasets, and the same datasets were applied to an MLR model as a benchmark for the random forest algorithm.	The work showed that the random forest algorithm is far more effective in crop yield prediction.

3	Liakos et. al. [2]	2018	This work involved a research into the use of machine learning agricultural production systems.	This work applied artificial neural networks.	This work showed that machine learning models have been used in several agriculture-related areas. Mainly in crop production and aiding management decision making processes.

4	Ming et. al. [5]	2016	This work involved classification of land cover based on image and remote sensing.	Random forest machine learning algorithm was used in the classification of image data.	Random forest is an efficient classification algorithm and performs effectively without using special selected features.

5	Nitze et al. [6]	2012	This work compared the effectiveness of several machine learning algorithms: support vector machine, artificial neural networks, and random forest.	several classifiers, Naïve Bayes for ML, random forest (RF), multilayer perceptron in case of ANN, and LibSVM for support vector machine, were used in this work for the classification of crops.	Even though classification results depended strongly on the number of images used, the SVM classifiers performed much better than the RF and ANN in most of the cases.

6	Chen and Cournede [7]	2017	This work focused on finding the most efficient way to predict the yield of corn based on meteorological records.	This work studied a new methodology named multiple scenarios parameter estimation and used the CORNFLO model.	Random forest regression was shown to be the most efficient for crop yield prediction.

7	Mitra et al. [8]	2017	This work focused on simulating and predicting crop yield for effective crop management and adequate results.	A three-layered artificial neural network (ANN) and R language were used in this work for prediction and simulation of crop yield.	The artificial neural network was effective for simulation and prediction.

**Table 2:** Final model distribution output table.

Class A	Class B	Class C
Cabbage, onion, tomato, peppers	Beans, corn, peas, sweet potato	Soya beans, carrot

## Data Availability

The data used to support the findings of this study are available from the corresponding author upon request. Ecological requirements are available in the following link: http://www.nafis.go.ke/agriculture/maize/ecological-requirements/.
